# Lycopene Modulates THP1 and Caco2 Cells Inflammatory State through Transcriptional and Nontranscriptional Processes

**DOI:** 10.1155/2014/507272

**Published:** 2014-05-07

**Authors:** Njock Makon-Sébastien, Fouchier Francis, Seree Eric, Villard Pierre Henri, Landrier Jean François, Pechere Laurent, Barra Yves, Champion Serge

**Affiliations:** ^1^Laboratoire de Génie Génétique, Faculté de Pharmacie, Aix-Marseille Université, 27 boulevard Jean Moulin, 13385 Marseille, France; ^2^Advanced Diagnostics, Toronto General Research Institute, University Health Network, 101 College Street, TMDT, Rm. 3-301, Toronto, ON, Canada M5G 1L7; ^3^INRA, UMR1260/1062 INSERM/AMU, Nutrition, Obésité et Risque Thrombotique, 27 boulevard Jean Moulin, 13385 Marseille, France; ^4^Laboratoire de Génie Génétique, INRA 1260, Faculté de Pharmacie, 27 boulevard Jean Moulin, 13385 Marseille Cedex 5, France; ^5^IMBE-UMR CNRS 7263/IRD 237, Mutagenèse Environnementale, Faculté de Pharmacie, Aix-Marseille Université, 27 boulevard Jean Moulin, 13385 Marseille, France; ^6^Laboratoires YVERY, 134 rue Edmond Rostand, 13008 Marseille, France

## Abstract

We revisited the action of a carotenoid, the lycopene, on the expression of proinflammatory genes, reactive oxygen species (ROS) production, and metalloprotease (MMP9) activity. THP1 and Caco2 cell lines were used as *in vitro* models for the two main cell types found in intestine tissue, that is, monocytes and epithelial cells. Proinflammatory condition was induced using either phorbol ester acetate (PMA), lipopolysaccharide (LPS) or tumor necrosis factor (TNF). In THP1 cells, short term pretreatment (2 h) with a low concentration (2 **μ**M) of lycopene reinforce proinflammatory gene expression. The extent of the effect of lycopene is dependent on the proinflammtory stimulus (PMA, LPS or TNF) used. Lycopene enhanced MMP9 secretion via a c-AMP-dependent process, and reduced ROS production at higher concentrations than 2 **μ**M. Cell culture media, conditioned by PMA-treated monocytes and then transferred on CaCo-2 epithelial cells, induced a proinflammatory state in these cells. The extent of this inflammatory effect was reduced when cells has been pretreated (12 h) with lycopene. At low concentration (2 **μ**M or less), lycopene appeared to promote an inflammatory state not correlated with ROS modulation. At higher concentration (5 **μ**M–20 **μ**M), an anti-inflammatory effect takes place as a decrease of ROS production was detected. So, both concentration and time have to be considered in order to define the exact issue of the effect of carotenoids present in meals.

## 1. Introduction

Epidemiological studies have shown that increased consumption of fruit and vegetables is associated with a lower risk of several kinds of pathologies [1–3]. Such beneficial effect could be due at least in part to the presence of carotenoids in the diet. Among carotenoids, lycopene has been particularly studied. Lycopene is a nonprovitamin A carotenoid found mainly in tomatoes and water melon. There is evidence suggesting that this compound plays a role in decreasing the occurrence of and the progression of certain cancers (prostate and hepatoma) [4–7] and several epidemiological studies have also linked high plasma lycopene concentrations to a lower risk of developing cardiovascular disease [4]. However, the mechanism underlying these effects remains unknown and there is debate over the impact of lycopene on human health [1–3].

It is well established that lycopene is able to modulate gene expression [8–12]. Several regulations have been depicted, including modulation of connexin 43 (Cx43) gene expression [13–15], inhibition of cell proliferation [15, 16], or induction of G1/S cell cycle arrest in the MCF-7 cell line by decreasing the expression of the D1 and D3 cyclins [17].

Furthermore, the properties of lycopene could rely on its transcriptional modulation of cytokine expression. It was notably demonstrated that lycopene and metabolites regulate cytokine expression in adipose tissue (suppress and adipocyte) [18–20]. In addition, lycopene was also suspected to act on the level of MMP9 expression [21], which is known to be involved in basal membrane degradation and this process plays a major role in inflammation [22, 23] and cell invasion [24].

Thus, regulation of inflammation appears as a common process connecting molecular effects of lycopene. However, only few studies have been especially devoted to this aspect in intestine, which constitute the first line of effect of micronutrients. Indeed, intestinal epithelial cells provide the first point of contact for micronutrients within the gut lumen; they also interface and segregate the gut immune system through which nutrients can trigger or modulate proinflammatory gene transcription, leading to an amplification of the inflammatory immune response [25, 26]. The currently available data derived from, respectively,* in vivo* and* in vitro* models appeared contradictory. Indeed, in mice models, it was found that lycopene-rich extract prevented lipopolysaccharide-induced NF*κ*B signaling [27]. It was also reported that lycopene supplementation attenuated the inflammatory status of colitis in a rat model and had a beneficial effect on the various macroscopic parameters examined including: colonic thickness, colon weight, and inflammation [28]. In contrast, it was showed that exposure of the CaCo-2 human cell line model to carotenoid had no statistical significant anti-inflammatory effect on IL-8, NO, COX2 expression, and regulation of NF*κ*B and MAPK activities [29].

In the present study, we investigated the effects of physiological concentration (2 *μ*M) of lycopene on monocyte and an intestine-derived epithelial cell lines, on various proinflammatory targets, that is, ROS production, transcription of cytokines, and MMP9 as well as MMP9 activities.

## 2. Methods and Materials 

### 2.1. Materials

RPMI 1640 supplemented with Hepes and glutamine was purchased from Life Technologies (Cergy-Pontoise, France). Foetal calf serum (FCS) (“HyClone,” lot n°CPB0054) was from Perbio (Thermo scientific, Brebières, France). The bicinchoninic acid (BCA) protein assay reagent was from Pierce (Interchim, Montluçon, France). The luciferase detection kit was from Promega France (Charbonnières. France). The following compounds were from Sigma (L'Isle d'Abeau, France): phosphate-buffered saline (PBS), bovine serum albumin (BSA), phenylmethylsulfonyl fluoride (PMSF), iodoacetamide, aprotinin, leupeptin, peptatin, o-phenanthroline, DEAE-dextran, 4-methyl-umbelliferyl-*β*-D-galactoside (MUG), phorbol myristate acétate (PMA), vitamin D3 (VD3), retinoïc acid (AR), lucigenin, lycopene, interferon *γ* (IFN*γ*), Tumor Necrosis Factor *α* (TNF*α*), N-(Cis-2-phenyl-cyclopentyl) azacyclotridecan-2-imine-hydrochloride (MDL-12,330A) and E. Coli-derived lipopolysaccharide 055:B5 (LPS), isobutyl-methylxanthine (IBMX), dibutyryl cyclic AMP (dbcAMP), and Forskolin (FSK). cAMP enzyme immunoassay detection kit was from “Assay Designs” (Stressgen, Euromedex, Strasbourg, France). The rabbit antiactive extracellular signal-regulated protein kinase (ERK) was from Promega France (Charbonnières, France).

### 2.2. Preparation of Lycopene Encapsulated in Gelatin Particles

Encapsulation of lycopene in gelatin particles was carried out according to the method described by Auweter [30]. Preliminary assays have demonstrated that this preparation displayed a better biological activity (x2) compared to lycopene dissolved in solvent. Biodisponibility of the lycopene encapsulated in gelatin was evaluated* in vitro* using Caco2 cells cultured on transfilter (Millicell, Millipore Corporation) and assessment of the transport of lycopene through the epithelial cells monolayer by spectrophotometric measurement of lycopene in the basal compartment. We found an enhancement of around 4 times of the biodisponibility of encapsulated lycopene compared to lycopene dissolved in solvent such as THF or DMSO. We found also over one year at 4°C a high stability of the dessicated and encapsulated product as indicated by spectrophotometry.

Bioactivity and biodisponibility of encapsulated lycopene were also compared to nanoemulsions of lycopene prepared using a mixture of Compritol ATo 888/Polysorbate 80/Soya lecithin. As such emulsion gives comparable results to lycopene encapsulated in gelatin and as gelatin appeared as a more neutral material than component used in emulsion, we decided to carry out all experiments with lycopene encapsulated in gelatin.

Lycopene-free gelatin particules were used as a control.

### 2.3. Cell Culture and Treatment

The intestinal epithelial Caco2 and the monocytic THP-1 human cell lines were routinely cultured in, respectively, DMEM and RPMI 1640 containing 2 mM glutamine, 50 U/mL penicillin, and 50 *μ*g/mL streptomycin and supplemented with 10% (v/v) FCS at 37°C in 5% CO_2_ atmosphere. The viability of cells was assessed by Trypan blue exclusion. In all the experiments, cells were used at a density of 1 × 10^6^ cells/mL.

Before the stimulation, cells were cultured for 2 h in the absence of FCS in a BSA-supplemented medium (0.2%) and, according to assays, with or without lycopene (2 *μ*M). Appropriate controls containing lycopene-free gelatin particles were carried out. Following this pretreatment, the cells were washed and a new identical medium supplemented or not with carotenoids was added with appropriate stimulator (PMA, 0.1 *μ*M; LPS 0.1 *μ*g/mL; TNF*α*, 10 ng/mL). The culture was resumed for 6 h and the cells were recovered, washed, and placed at −80°C. mRNA expression was analyzed by RT-QPCR.

### 2.4. Plasmids

The regulatory sequences harbouring AP1-binding sequence and NF*κ*B-binding sequence were derived from, respectively, the MMP9 (kindly supplied by Dr. Boyd) [31, 32] and IL-8 and IgK gene enhancer regions which have been already described [33–36]. These regulatory sequences were introduced in pGL2-based plasmids (Promega) bearing an intrinsic promoter and the luciferase reporter gene.

### 2.5. Cell Transfection and Stimulation

THP1 cells were transiently transfected using DEAE-dextran-based method according to Aneja et al. [26] in STBS buffer (25 mM Tris-HCl, pH 7.4, 137 mM NaCl, 5 mM KCl, 0.6 mM Na_2_HPO_4_, 0.7 mM CaCl_2_, and 0.5 mM MgCl_2_).

A total of 25 × 10^6^ cells were cotransfected for 20 min in 0.5 mL STBS containing 200 *μ*g DEAE- dextran and 0.5 *μ*g plasmid to be tested together with 0.5 *μ*g of the pCMV plasmid expressing *β*-galactosidase under the control of the CMV promoter (pCMV-*β*-gal). Then the cells were washed, resuspended in 12 mL RPMI/10% FCS, and incubated for 24 h. For assays, the cells were washed and resuspended in 25 mL RPMI/0, 2% BSA and distributed in 6-well plates (2 mL/well). Then, according to assays, lycopene (2 *μ*M) was added and 2 h after other agonists (TNF*α*, 10 ng/mL or PMA, 0.1 *μ*M) to be tested. Incubation resumed for indicated time. Cell extracts were performed using the luciferase kit lysing buffer (Promega) following the manufacturer's instructions, and protein content was determined using the micro BCA reagent.

### 2.6. *β*-Galactosidase Assay

*β*-galactosidase activity was determined using the 4-methyl-umbelliferyl-*β*-D-galactoside (MUG). Each sample (30 *μ*g proteins) was incubated for 1 hour at 37°C in a reaction mixture (100 *μ*L) containing 6 mM Na_2_HPO_4_, 4 mM NaH_2_PO_4_, 10 mM KCl, 0.1 mM MgSO_4_, 50 mM *β*-mercaptoethanol, and 0.5 mM MUG, pH 7. The reaction was stopped by adding 400 *μ*L of 100 mM glycine pH 11. Fluorescence of the product was recorded using a Perkin Elmer fluorometer, setting excitation light at 360 nm and emission at 446 nm. Values of the *β*-galactosidase-induced fluorescence were used to normalize the luciferase activity for transfection efficiency.

### 2.7. Luciferase Assay

Luciferase assays were performed using 30 *μ*g proteins of the cell extracts with the luciferase assay reagent, according to the instructions of the manufacturer (Promega) and using a luminometer (Berthold, France SAS). Results were reported as arbitrary light units of luciferase normalized relatively to the *β*-galactosidase activity.

For transfection, each assay was performed in triplicate. Total experiments were repeated twice at least and results from a representative experiment were shown.

### 2.8. RNA Extraction and Real-Time PCR

Total RNA was isolated from the cells using Trizol reagent (Invitrogen, France). RNA content was quantified by optical density measurement at 260 nm using NanoDrop device (NanoDrop technologies Inc., France). 4 *μ*g of RNA was reverse-transcribed into cDNA using Moloney virus-derived reverse transcriptase (Invitrogen, Cergy-Pontoise, France) and random primers at 42°C for 1 h. The expression of target genes was determined using the Stratagene System (MX3005P) (Stratagene, France). PCR was performed with 0.5 *μ*M of each primer, 0.5 *μ*M of the gene specific UPL Probe (Roche), and the LightCycler Taqman and 5X Master MIX Plus (Roche), in a total volume of 10 *μ*L. Cycling conditions were as follows: 2 min at 40°C, 10 min denaturing at 95°C, followed by 40 cycles of 10 s denaturing at 95°C, 30 s primer annealing at 60°C, and 10 s fragment elongation at 72°C. The melting curve was analyzed with the LightCycler 480 and MXPRO gene scanning softwares. Human interleukin-1 *β* (IL-1*β*), interleukin-8 (IL-8), intercellular adhesion molecule-1 (ICAM-1), and metalloprotease-9 (MMP-9) expressions were normalized to *β*2-microglobulin expression and data quantified by the method of 2^−ΔΔCt^ [37]. The primers  used are listed in Table 1.

### 2.9. Preparation of Conditioned Medium (CM) Derived from PMA-Treated THP1 Cells and Assay on Caco2 Cells

In order to study the cross talk between inflammatory cells (THP1) and intestinal cells (CaCo-2), THP1 conditioned medium (CM) was prepared. THP1 cells were exposed or not to PMA for 2 hours. The medium was then discarded and the cells were extensively washed. Fresh medium without PMA was added for an additional 6 h incubation. At the end of this incubation, the media conditioned by THP1 cells were collected and then added for 6 h to Caco2 cell cultures pretreated or not with lycopene for 12 h. The expression of inflammatory genes was assessed on these cell cultures.

### 2.10. Assay for Lycopene-Mediated Increase in cAMP Production

Cultured cells were washed with prewarmed PBS and resuspended with cell suspension media (RPMI 1640 containing 50 mM HEPES, pH 7.4) at 37°C. Cells were pretreated with PDE inhibitor (50 *μ*M IBMX) for 15 min and then treated for different times (from 5 to 30 min) with lycopene (2 *μ*M) as indicated. Treatments were terminated by addition of 1 N HCl (0.1 N HCl final). Total cAMP (intra- and extracellular) was detected by enzyme immunoassay. Data represent mean ± SD with *n* = 8.

### 2.11. Zymography

THP1 cells were incubated for 12 h in the serum-free medium supplemented or not with lycopene and other compounds to be tested. The medium was then recovered and clarified by centrifugation (3000 g, 15 min) and tested by zymography for proteolytic activity.

Zymography was performed using 10% PAGE containing gelatin (1 mg/mL). Conditioned medium was loaded on the gel (5−20 *μ*L) and migration was performed at 4°C for 2 h, 15 min at 120 volts. After electrophoresis, gels were washed twice in buffer containing (50 mM Tris, pH 7.5; 0.15 M NaCl; 2.5% Triton X100) for 30 min to remove SDS. Proteolytic activity was revealed by incubation of the gel in the same buffer without Triton but supplemented with appropriate concentrations of cations (5 mM Ca^2+^, 0.5 mM Mn^2+^). After fixation and coloration (Coomassie blue), enzyme activity was identified as clear bands against the blue background. Gel was scanned and the digitized picture was further contrasted using PaintShop Pro software. Cell treatment and zymography were repeated at least three times and results from a representative experiment were shown.

### 2.12. ROS Determination

The ability of lycopene to modify ROS production was tested on macrophages-derived THP1 cells treated with PMA and was recorded by lucigenin-induced luminescence.

Differentiation of THP1 cells was carried out by supplementation of the culture medium with AR (1 *μ*M), VD3 (0.1 *μ*M), and IFN*γ* (10 U/mL = 5 ng/mL) for 48 h. Obtained macrophages were or not treated with lycopene (0.5–20 *μ*M) during 6 hours and cell monolayer was scratched and suspended in a RPMI-based medium without phenol red supplemented with FCS (10%). Then, the suspension was distributed in an opaque 96-well plate (200 *μ*L in each well- 0.7 × 10^5^ cells). Lucigenin solution (25 *μ*L) (51 mg lucigenin, 476 mg Hepes, and 100 mg gelatin/100 mL RPMI) and, according to assays, PMA (200 nM final concentration, 25 *μ*L) was added. Three assays were carried out for each condition tested.

Luminescence was immediately recorded in the “luminoscan” system. Intensity values of luminescence were recorded each 5 min during a total period of 60 min. Graphs were constructed and integration of curves was carried out using the excel software. Values were plotted as relative units.

### 2.13. Statistical Analysis

Statistical analysis was performed using GraphPad Prism Software. Mean ± SD and intergroup comparisons were, respectively, obtained with ANOVA and Tukey's test. Values were considered statistically different when *P* < 0.05. Results are presented as means ± SD.

## 3. Results and Discussion

### 3.1. Results

#### 3.1.1. Lycopene Modulates Proinflammatory Cytokine Expression in THP-1

**Figure 1:**
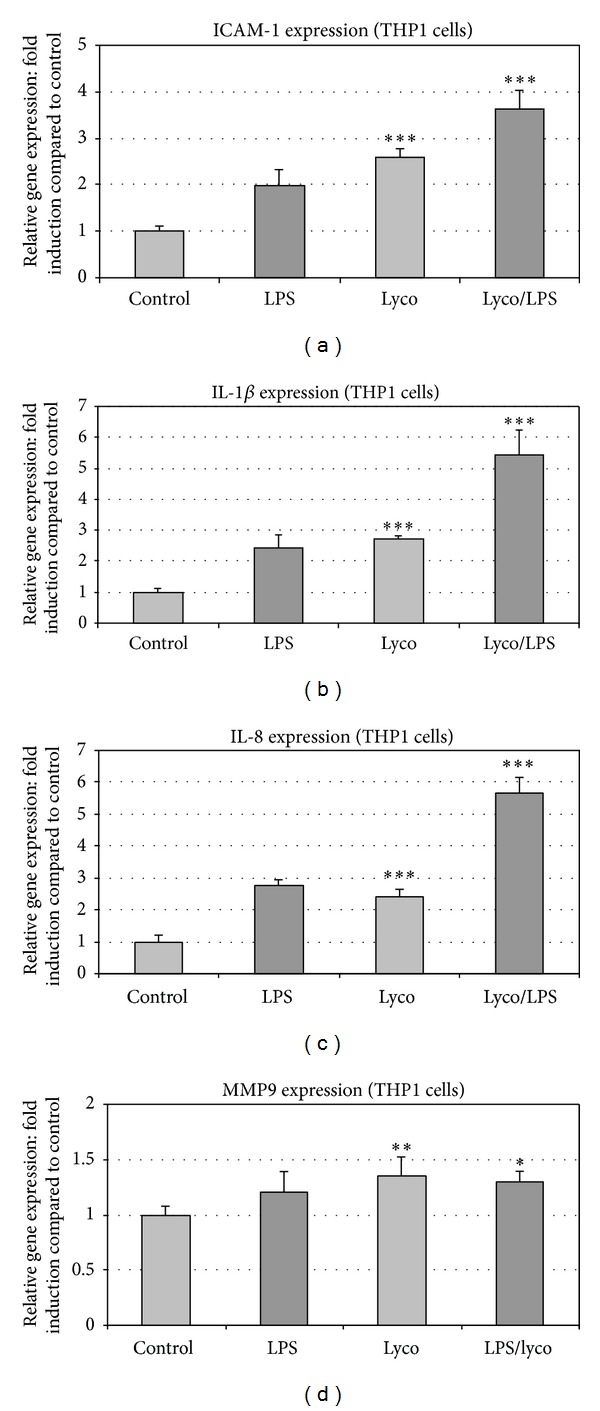
Lycopene modulates basal and LPS-induced inflammatory gene expressions in THP1 cells. ICAM-1 (a), IL-1*β* (b), IL-8 (c), and MMP9 (d) gene expression was studied in THP1 cells treated with LPS. Modulation of this expression was studied in THP1 cell cultures pretreated for 2 h with or without lycopene (2 *μ*M). Then, according to assays, LPS (100 ng/mL) was added for 6 h and mRNA expression was analyzed by RT-qPCR. Values are means ± SD (*n* = 8). Assays treated with lycopene were compared to corresponding assays without lycopene. ***P < 0.01; **P < 0.05; ∗, nonsignificantly different.

The modulation of expression of several genes linked to inflammation (ICAM-1, IL-1*β*, IL-8, and MMP9) was studied on THP1 cell cultures (Figure 1).

Lycopene enhanced the basal expression of proinflammatory genes, ICAM-1, IL-1*β*, and IL-8, and further enhanced the LPS-induced gene expression (Figures 1(a), 1(b), and 1(c)). MMP9 gene expression was only slightly modified in the presence of lycopene (Figure 1(d)). Preliminary assays have shown that the lycopene concentration used in this report (2 *μ*M) was the lower one displaying an efficient activity on gene transcription with no effect on ROS production.

**Figure 2:**
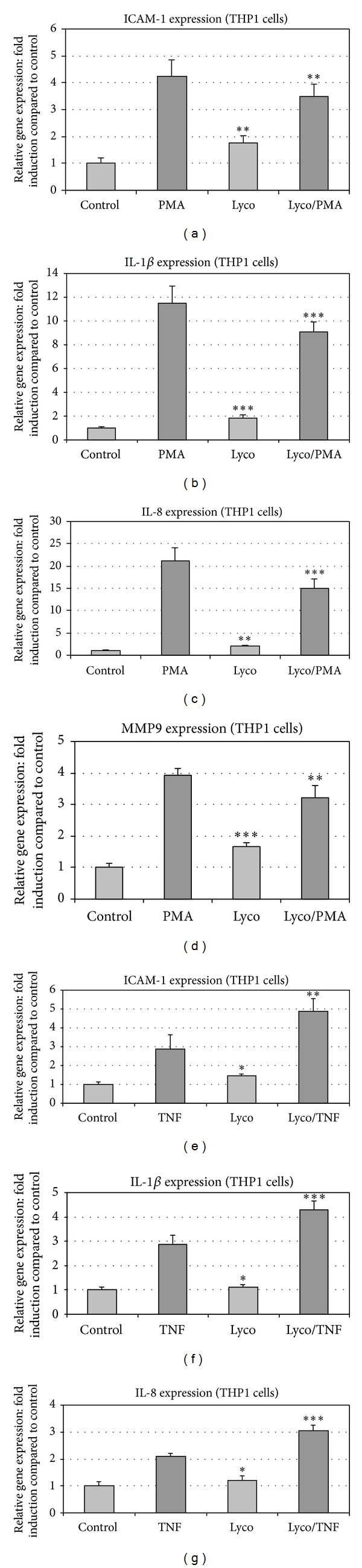
Lycopene modulates gene expression induced by PMA and TNF in THP1 cells. ICAM-1 ((a), (e)), IL-1*β* ((b), (f)), IL-8 ((c), (g)), and MMP9 (d) gene expression was studied in THP1 cells treated with either PMA (a,b,c, and d) or TNF*α* ((e), (f), and (g)). Modulation of this expression was studied in THP1 cell cultures pretreated for 2 h with or without Lycopene (2 *μ*M). Then, according to assays, PMA (100 nM) or TNF*α* (10 ng/mL) was added for 6 h and mRNA expression was analyzed by RT-qPCR. Values are means ± SD (*n* = 8). Assays treated with lycopene were compared to corresponding assays without lycopene. ***P < 0.01; **P < 0.05; ∗, nonsignificantly different.

The same type of experiment was conducted with PMA as inflammatory stimulating molecule. Incubation of THP1 cells with PMA strongly enhanced the expression of inflammatory gene (Figures 2(a), 2(b), 2(c), and 2(d)). The PMA-induced enhancement of gene expression was reduced by around 30% in cells pretreated 2 h with lycopene (Figure 2). In contrast to LPS, PMA induced strongly MMP9 expression, and this enhancement was slightly reduced in cells pretreated with lycopene (Figure 2(d)).

Finally, TNF*α*, a third proinflammatory molecule used, upregulated gene expression of ICAM1, IL-1*β*, and IL-8, and lycopene enhanced this effect (Figures 2(e), 2(f), and 2(g)).

These data suggested that the change in cytokine gene expression induced by lycopene was gene dependent but was also dependent on the nature of the proinflammatory stimulus (PMA, LPS, or TNF*α*).

#### 3.1.2. Lycopene Modulates NF*κ*B and AP-1 Transactivation in THP-1

The possible lycopene-induced modulation of transcriptional response to cytokine was tested using reporter gene assay. Due to the induced high mortality in the transfected THP1 cells after LPS treatment, we used TNF*α* and PMA as stimuli to enhance gene reporter expression in transfected THP1 cells since these proinflammatory molecules did not induce apoptosis in the transfected cells (data not shown).

**Figure 3:**
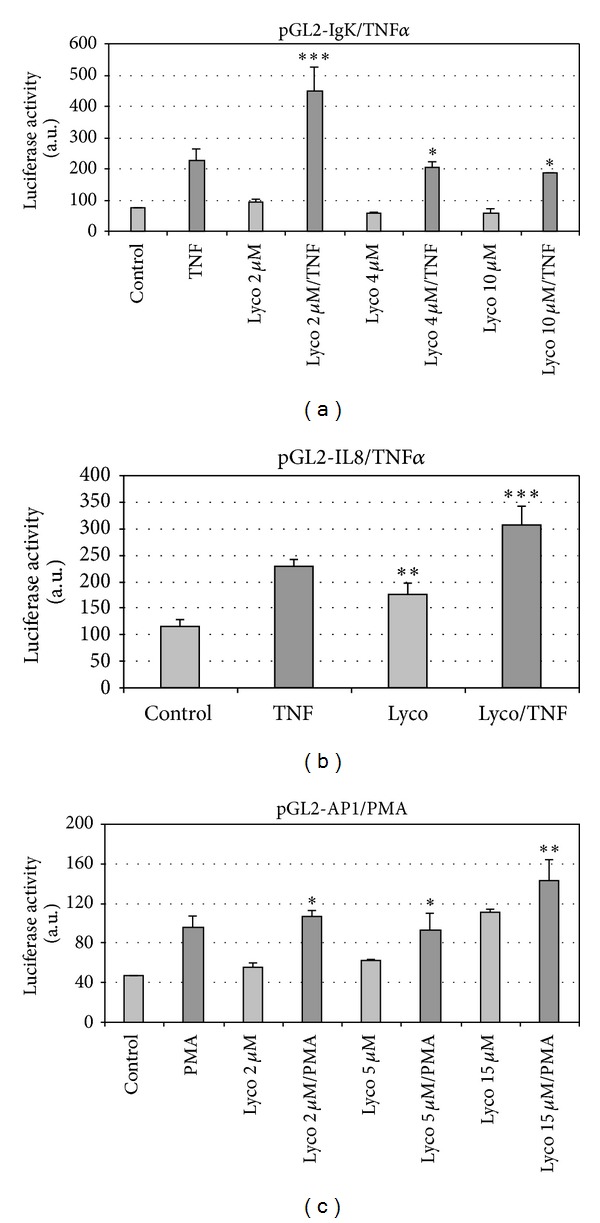
Modulation by lycopene of reporter gene activity in THP1 cells. THP1 cells were transfected using one of the luciferase reporter genes controlled by the promoter/enhancer sequence derived from either the IgK gene (pGL2-IgK-luc) (a) or the IL-8 gene (pGL2-IL-8-luc) (b). The plasmid pGL2-AP1-luc (c) contains the AP1 specific sequence. After 12 h transfection, cells were washed and fresh medium with or without lycopene at the indicated concentration was added, and culture continued for 2 h. Then, according to assays, TNF*α* ((a), (b)) or PMA (c) was added and culture was resumed for 6 h. Cells were harvested, lysed, and placed at −80°C. Luciferase activity was recorded. Values are means ± SD (*n* = 8). Assays treated with lycopene were compared to corresponding assays without lycopene. ***P < 0.01; **P < 0.05; ∗, nonsignificantly different.

Reporter gene assays (Figures 3(a) and 3(b)) showed that the lycopene-induced enhancement of cytokine-stimulated gene expression involved NF*κ*B-binding sites. Indeed, in the assays, reporter gene was driven by promoter/enhancer sequence derived from both IgK and IL-8 genes which include at least one NF*κ*B-response element. TNF*α*-stimulated reporter gene expression was enhanced with 2 *μ*M lycopene (Figures 3(a) and 3(b)), whereas higher concentrations (4 and 10 *μ*M) were ineffective compared to TNF*α* (Figure 3(a)). Reporter gene placed under the control of AP1 (PK3-CAT plasmid contains three AP1-binding sites but no NF*κ*B-binding site) was responsive to PMA and lycopene displayed a positive effect on this response only at 15 *μ*M (Figure 3(c)).

#### 3.1.3. ROS Are Not Involved in the Regulation of Cytokine Expression

**Figure 4:**
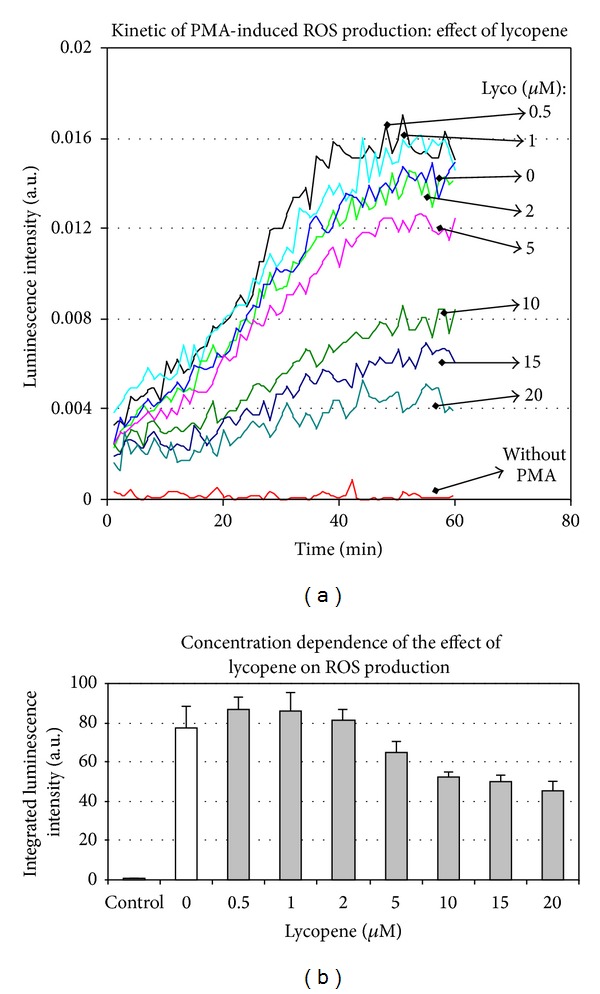
Lycopene modulates PMA-induced ROS production in THP1 cells. THP1 cell suspension was distributed into multiwell plates and, according to the assays, different concentrations of lycopene were added for 2 hours. Then, PMA (100 nM) together with lucigenin was added, and ROS-induced luminescence was immediately recorded for 60 min (a). Data were collected on computer and kinetics integration was performed (b). A representative experiment is shown. Values are means ± SD (*n* = 8).

We investigated the contribution of antioxidant effect of lycopene in the depicted regulation. The transcriptional effects of lycopene appeared not to be linked to its antioxidant properties. Indeed, as tested by luminescence on THP1-derived macrophages recording, only lycopene concentrations above 5 *μ*M were effective in reducing PMA-induced ROS production (Figures 4(a) and 4(b)).

#### 3.1.4. Lycopene-Induced Cytokine Expression and Modulation in Caco2 Cells

**Figure 5:**
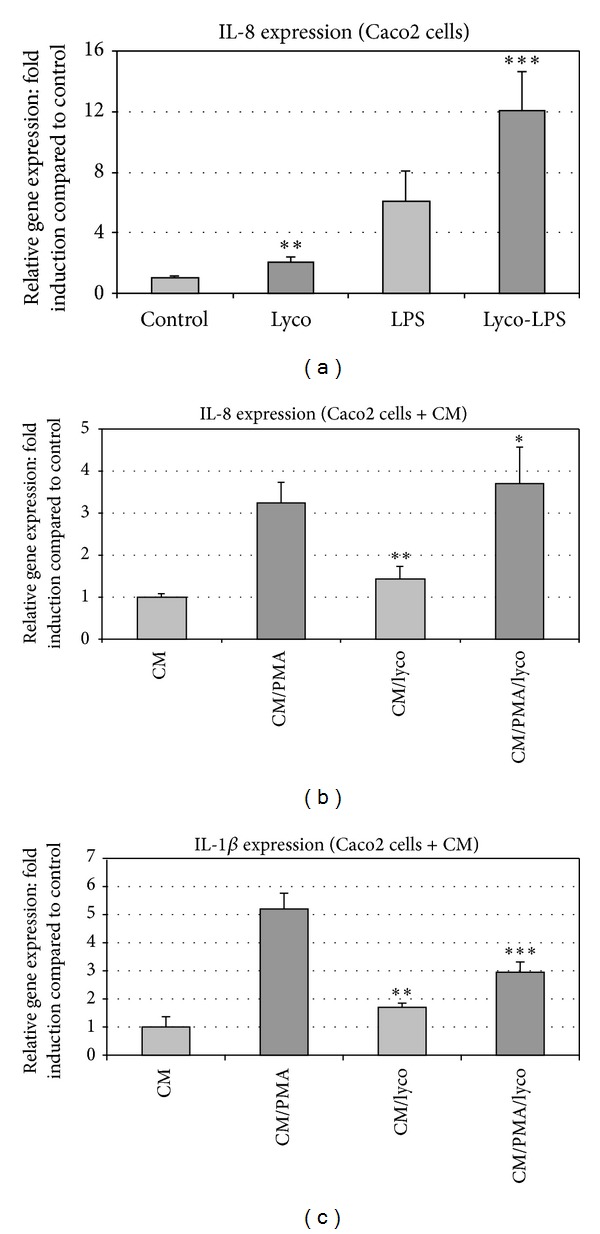
Lycopene-induced modulation of IL-8 and IL-1 gene expression in Caco2 cells. (a) Lycopene induced upregulation of basal and LPS-induced IL-8 gene expression in Caco2 cells. IL-8 gene expression was studied in Caco2 cells treated with LPS. Modulation of this expression was studied in Caco2 cell cultures pretreated for 2 h with or without vectorized lycopene. Then, according to assays, LPS (100 ng/mL) was added for 6 h and mRNA expression was analyzed by RT-qPCR. (b) Lycopene induced no modification of gene expression induced by THP1-derived conditioned medium (CM) in Caco2 cells. THP1 cells were or not exposed to PMA for 2 hours. Then, the cell culture medium was discarded and the cells were extensively washed and a fresh medium without PMA was added and culture was resumed for 6 h. At the end of the culture period, the conditioned media (CM) were collected and then added to Caco2 cell cultures pretreated or not with lycopene for 12 h. The final treatment of Caco2 cells with the conditioned medium (CM) was for 6 h and, finally, expression of inflammatory genes was assessed by qRT-PCR. (c) Lycopene downregulates IL-1 gene expression induced by THP1-derived conditioned medium (CM) in Caco2 cells. THP1 cells were or not exposed to PMA for 2 hours. Then, the cell culture medium was discarded and the cells were extensively washed and a fresh medium without PMA was added and culture was resumed for 6 h. At the end of the culture period, the conditioned media (CM) were collected and then added to Caco2 cell cultures pretreated or not with lycopene for 12 h. The final treatment of Caco2 cells with the conditioned medium (CM) was for 6 h and, finally, expression of inflammatory genes was assessed by qRT-PCR. Values are means ± SD (*n* = 8). Assays treated with lycopene were compared to corresponding assays without lycopene. ***P < 0.01; **P < 0.05; ∗, nonsignificantly different.

It is well known that the cytokine expression and inflammatory gene expression, in general, are upregulated in intestinal epithelial cells in response to various inflammatory signals [25]. In agreement, IL-8 expression was induced in Caco2 cells in response to LPS (Figure 5(a)). This expression was enhanced by lycopene, and lycopene alone also induced IL-8 expression (Figure 5(a)).

To study the possible transfer of inflammatory signals from monocytes/macrophages to epithelial cells, THP1 cell-derived conditioned media (CM) were prepared and tested on Caco2 cells pretreated or not with lycopene for 12 h, as described under materials and methods. The expression of inflammatory genes was assessed on these cell cultures.

In order to study the cross talk between inflammatory cells (THP1) and intestinal cells (CaCo-2), THP1 conditioned medium (CM) was used. This medium induced a marked enhancement of both IL-1*β* and IL-8 productions in Caco2 cells (Figures 5(b) and 5(c)). The IL-1*β* but not IL-8 enhancement was strongly reduced (by around 50%) in the lycopene-pretreated Caco2 cells.

#### 3.1.5. Lycopene Modulates MMP9 Activity via c-AMP

**Figure 6:**
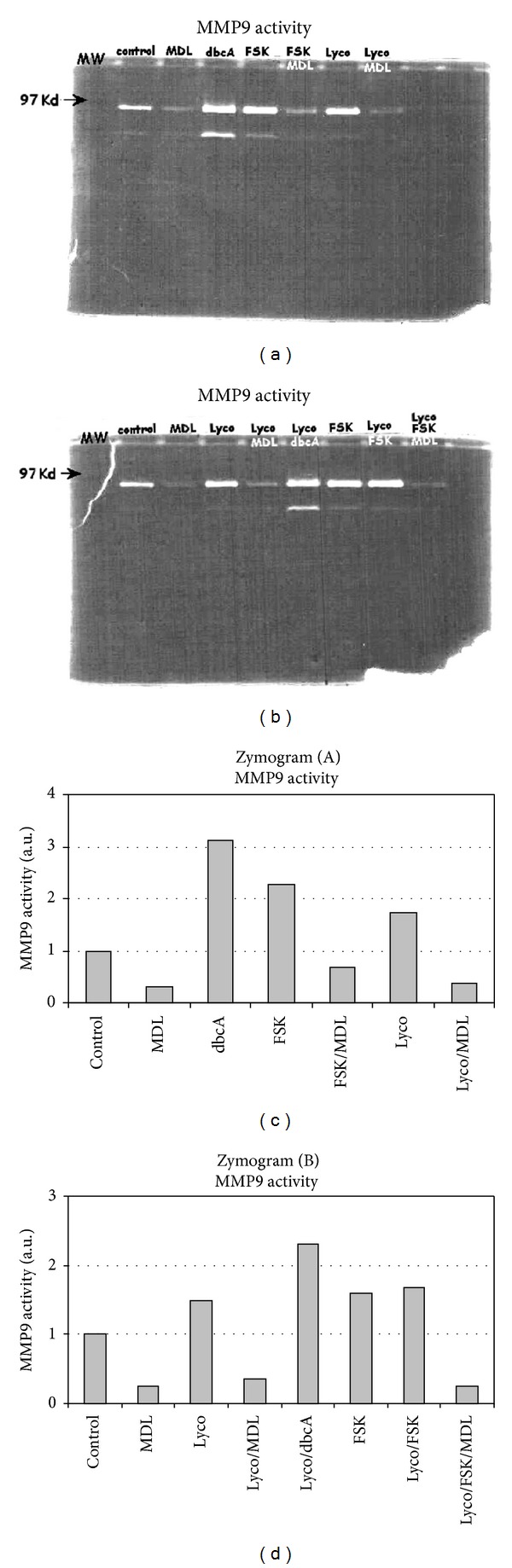
MMP9 activity is induced by lycopene in THP1 cells. THP1 cells were cultured for 12 h in the presence or not of lycopene (2 *μ*M) together with or without different effectors acting specifically on the c-AMP metabolism (FSK, MDL, and IBMX). Culture medium was collected and zymography ((a), (b)) was performed as described under Materials and Methods. Densitometry analysis of zymograms (A) and (B) was performed and results are reported on graphs (c) and (d), respectively. One representative experiment out of three is shown.

Lycopene enhanced the MMP9 activity in THP1-derived conditioned medium (Figures 6(a), 6(b), 6(c), and 6(d)). This effect did not appear to be dependent on an upregulation of gene transcription. Indeed, as reported above (Figure 1), and in contrast with an increase in enzymatic activity, lycopene did not trigger an enhancement of either basal or PMA-induced MMP9 mRNA expression in THP1 cells.

**Figure 7:**
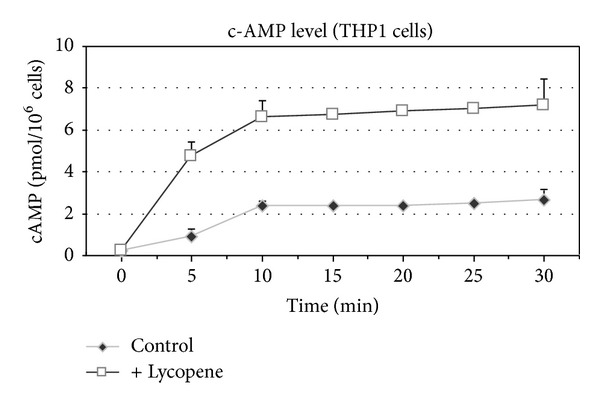
Lycopene-induced enhancement of c-AMP level in THP1 cells. THP1 cells were cultured for indicated times in the presence or not of lycopene (2 *μ*M). The cells were then harvested and analyzed for c-AMP content as indicated under Materials and Methods. Values are means ± SD (*n* = 6).

We next investigated the molecular basis of the lycopene-induced enhancement of MMP9 activity. The lycopene-induced enhancement of MMP9 gene transcription (Figure 2(d)) is significant but the effect appeared rather scarce compared to the enhancement of MMP9 enzyme activity (Figure 6). So we hypothesize that a posttranslational mechanism could be the main cause of the lycopene-induced enhancement of MMP9 activity. It has long been established that c-AMP is a regulator of various secretion processes, so we hypothesize that c-AMP may be involved in the lycopene-induced enhancement of MMP9 activity. Indeed, we found that the MMP9 secretion was upregulated by a range of compounds known to enhance the intracellular c-AMP concentrations; addition of forskolin (FSK) or dbcAMP to THP1 cell cultures treated or not with lycopene enhanced the activity of conditioned media-derived MMP9 (Figures 6(a) and 6(c)). These compounds did not modify MMP9 mRNA expression (data not shown). Furthermore, MDL-12,330A (MDL) which is known as a specific adenylate cyclase inhibitor, strongly reduced both basal and lycopene-enhanced MMP9 secretions (Figures 6(a), 6(b), 6(c), and 6(d)) and a low concentration of lycopene (2 *μ*M) induced a time-dependent enhancement of c-AMP production in IBMX-pretreated THP1 cells (Figure 7).

### 3.2. Discussion

In the present study we reported the effect of lycopene on the expression of genes involved in inflammation, in intestinal epithelial cells and monocytes in culture. To this purpose, the effect of lycopene was studied* in vitro* using Caco2 and THP1 cells. In addition, interactions between intestinal epithelial and immune cells were envisioned through experiments involving conditioned medium.

Considering the expressions of IL-1*β*, ICAM, and IL-8, we found that, according to the kind of the cell and stimulus, a low concentration of lycopene induced two opposite effects on gene expression. We observed an acute and direct upregulation of either basal or LPS- and TNF*α*-stimulated levels in THP1 cells but a downregulation of the PMA-enhanced expression of these genes. On epithelial Caco2 cells stimulated by a conditioned medium, lycopene induced also a dowregulation on IL1*α* expression but had no effect on IL8 expression. These results suggested that there are at least two different targets for the lycopene at the gene regulation level, leading to either enhanced or decreased expression of the proinflammatory genes.

Present results on both gene and ROS expressions let us exclude a possible mechanism of control of gene expression triggered by low concentration of lycopene involving a modification of ROS production. Indeed, such a low concentration of lycopene did not modify ROS production in THP1-derived macrophages and consequently it appears that, at least in short term cell incubation and only considering this low lycopene concentration, ROS were not involved in the lycopene-induced gene regulation.

The pathway involving ROS has been suggested to be an important process involved in some but not all carotenoid-induced effects [14, 15]. However, this work makes unlikely the assumption according to which a single pathway involving ROS modulation could support all the actions of lycopene, especially on NF*κ*B (or AP1) activity. This meaning was corroborated by assays using reporter gene placed under the control of IgK enhancer bearing NF*κ*B binding sites; these assays showed that only a low lycopene concentration leads to an enhancement of TNF*α*-induced reporter gene expression but this effect disappeared in the presence of a higher concentration of lycopene.

It is noteworthy that present experimentation has been performed with “physiological” concentration of lycopene, corresponding to a serum concentration observed following a moderate consumption of this carotenoid. Indeed, in a range of studies, the main concern is about the exact bioactive concentration present in the organism and the real cell bioavailability of this highly lipophilic compound [38, 39] and, in some* in vitro* studies, the high concentration of lycopene used (10 *μ*M or more) may preclude possible physiological and nutritional interpretations of the results.

The gene induction triggered in Caco2 cells in response to a THP1 cell culture-derived conditioned medium showed that the inflammation induced in immune cells was transferable to epithelial cells. We found that whatever the range of induction is only a certain proportion (around 50%) of gene expression was found to be reduced in cells pretreated with lycopene, showing that this compound probably modulates only one of the pathways involved in gene induction.

In this work, we have shown that an important yet previously unrecognized second level of regulation could be operative in monocytes. Indeed, MMP9 secretion was modulated in the presence of lycopene, and the c-AMP/PKA system appears to be involved in this process.

A range of observations reported in the literature could support these data. Indeed, it is known that traffic of MMP-containing vesicles and exocytosis is dependent on microtubules [40] and that c-AMP is known to be involved in various secretion mechanisms via the regulation of exocytosis in many cells [41–44]. Furthermore, it is established that c-AMP controls cell proliferation [45–47] and it has been reported that a moderate activation of cAMP/PKA is sufficient to enhance the chronic inflammation [48]. Finally, adenylate cyclase, c-AMP, and PKA have all been shown to be involved in some of the effects of carotenoids [46, 47].

Cell treatment with lycopene leads to enhanced c-AMP cell content. Given that this enhancement was amplified by the addition of IBMX and that the increase in lycopene-induced MMP9 activity was prevented by cell cotreatment with an inhibitor of c-AMP production, we concluded that the action of lycopene involved c-AMP synthesis. This interpretation is further supported by our present data showing that compounds known to induce enhanced c-AMP synthesis (dbcAMP and Forskolin) also enhance MMP9 secretion as illustrated by the activity levels found in conditioned media derived from cells treated with these compounds.

What is the mechanism that enables lycopene to induce c-AMP synthesis? An answer could involve a mechanism modulating the coupling of adenylate cyclase with G proteins. G proteins tend to cluster in specialized distinct microdomains [49, 50] and remain confined to caveolae structures [50]. For example, disassembly of caveolae structure with cyclodextrin led to enhanced c-AMP accumulation in response to *β*2-adrenergic receptor agonists [50]. Thus, the interaction of G*α* protein subunits, and adenylate cyclase enzymes, is dampened by the interaction with caveolin protein (cav-1) which is the main constituent of caveolae.

It was reported that carotene at low concentration acted as a growth-inhibiting agent in cav-1-positive cells but not in cav-1-negative cells and regulated the expression of cav-1 protein [50], showing that carotene may modulate cell activity through a mechanism involving cav-1 protein. This modulation could involve disassembly of caveolae structure [50] and consequently enhanced adenylate cyclase and c-AMP level. The fact that caveolin is expressed in THP1 cells makes it possible that, in this cell line, carotenoids worked via a mechanism involving caveolin and c-AMP [51].

## 4. Conclusions

The experimental conditions used in this work were aimed at reproducing a proinflammatory state of the intestine during which reactive monocytes are recruited. We conclude that a low carotenoid concentration could modulate physiological and pathological proinflammatory conditions through both transcriptional and nontranscriptional actions, independently of ROS production. This low concentration may influence the enhancement of inflammation induced by a cross talk between epithelial and newly recruited monocytes in the gastrointestinal mucosa.

## Figures and Tables

**Table 1:** Sequence of “UPL” primers used for RT-qPCR.

Genes	Forward sequence (5′-3′)	Reverse sequence (5′-3′)	UPL Probe *n*°
ICAM	5′-CCTTCCTCACCGTGTACTGG-3′	5′-AGCGTAGGGTAAGGTTCTTGC-3′	Number 71
IL-8	5′-AGACAGCAGAGCACACAAGC-3′	5′-ATGGTTCCTTCCGGTGGT-3′	Number 72
TGF*β*	5′-CCGGATACTCACGCCAGA-3′	5′-AGAGATACGCAGGTGCAGGT-3′	Number 28
TNF*α*	5′-CAGCCTCTTCTCCTTCCTGA-3′	5′-GCCAGAGGGCTGATTAGAGA-3′	Number 29
MMP9	5′-TGTACCGCTATGGTTACACTCG-3′	5′-GCCCCAGAGATTTCGACTC-3′	Number 53
IL-1*β*	5′-AACAGGCTGCTCTGGGATT-3′	5′-TGGCTGCTTCAGACACTTGA-3′	Number 41
*β*2-microgl	5′-TTCTGGCCTGGAGGCTATC-3′	5′-TCAGGAAATTTGACTTTCCATTC-3′	Number 42
